# Ribavirin Treatment for Severe Schizophrenia with Anti-Borna Disease Virus 1 Antibodies 30 Years after Onset

**DOI:** 10.1155/2023/4899364

**Published:** 2023-02-27

**Authors:** Hidenori Matsunaga, Akio Fukumori, Kohji Mori, Takashi Morihara, Shunsuke Sato, Kyoko Kitauchi, Kanta Yanagida, Kazumi Taguchi, Tomoyuki Honda, Keizo Tomonaga

**Affiliations:** ^1^Department of Psychiatry, Osaka General Medical Center, Osaka, Japan; ^2^Department of Psychiatry, Osaka University Graduate School of Medicine, Suita, Osaka, Japan; ^3^Department of Pharmacotherapeutics II, Faculty of Pharmacy, Osaka Medical and Pharmaceutical University, Takatsuki, Osaka, Japan; ^4^Department of Mental Health Promotion, Osaka University Graduate School of Medicine, Toyonaka, Osaka, Japan; ^5^Department of Psychiatry, Toyonaka Municipal Hospital, Osaka, Japan; ^6^Department of Virology, Graduate School of Medicine, Dentistry and Pharmaceutical Science, Okayama University, Okayama, Japan; ^7^Laboratory of RNA Viruses, Department of Virus Research, Institute for Frontier Life and Medical Sciences, Kyoto University, Kyoto, Japan

## Abstract

**Objective:**

Borna disease virus 1 (BoDV-1) was proven to cause fatal encephalitis in humans in 2018. However, the effects of persistent infections remain unclear. Here, we present the case of a 50-year-old woman with a 30-year history of severe schizophrenia, who was exposed to fleas from stray cats prior to disease onset, suggesting the possibility of zoonosis including BoDV-1 infection. The patient had experienced significant social impairment, thought deterioration, delusions, and hallucinations for more than 20 years.

**Method:**

A radioligand assay was used to test the patient for IgG and IgM antibodies against BoDV-1 nucleoprotein (N) and phosphoprotein (P). Based on the protocol for hepatitis C, we treated the patient with 400 mg/day ribavirin, which was later increased to 600 mg/day.

**Results:**

The serological examination revealed anti-BoDV-1 N IgG. Although only subtle changes were observed over the 24 weeks of treatment, the family noticed that the patient's Cotard delusions had disappeared 7 months after completing the treatment, accompanied by some improvements in the relationship with the family.

**Conclusion:**

Though definite proof was not obtained, this presumed suppression of BoDV-1 by ribavirin leading to improvements in Cotard syndrome-like symptoms suggests that intractable schizophrenia might be one of the BoDV-1 infection phenotypes. Further studies are needed to clarify the effect of persistent BoDV-1 infections in humans.

## 1. Introduction

Although the precise biological etiology of schizophrenia remains largely unexplored, it is currently considered to be a multifactorial syndrome with varying etiologies and presentations. Recent studies have focused on immune dysfunction in the brains of schizophrenia patients [1, 2]. Additionally, several genome-wide studies have reported an association between schizophrenia and the human leukocyte antigen locus, strongly suggesting a genetic role of immunity in the development of schizophrenia [3, 4]. Regarding the infectious disease hypothesis, a meta-analysis conducted by Arias et al. that thoroughly analyzed a variety of infectious agents revealed that six out of 20 microbes were significantly associated with schizophrenia, including human herpes virus 2, human endogenous retrovirus W, *Chlamydophila pneumoniae*, *Chlamydophila psittaci*, *Toxoplasma gondii*, and Borna disease virus (BoDV-1) [5]. Two other meta-analyses found that *Toxoplasma gondii* and BoDV-1 are significantly associated with schizophrenia [6, 7].

BoDV-1 has been studied in veterinary medicine in Germany as a cause of meningoencephalitis in horses. It is a negative-stranded RNA virus that infects a wide range of vertebrates including cattle, sheep, rodents, primates, dogs, cats, and horses [8]. Not only does it cause acute fatal encephalitis, but persistent noninflammatory infections also lead to behavioral abnormalities [9]. In 1985, antibodies specific to BoDV-1 were found in the sera of patients with affective disorders [10], leading to the investigation of a possible causal relationship with human psychiatric diseases.

Bechter et al. conducted a large epidemiological study using indirect immunofluorescence assays and found that although disease specificity among psychiatric groups was not observed, younger patients with psychiatric diseases had significantly higher rates of antibodies against BoDV-1 (psychiatric, neurological, and surgical patients, 6.02%, 3.7%, and 2.2%, respectively) [11]. When comparing psychiatric patients with and without antibodies, the positive group included more patients with first-episode schizophrenia without complete remission, as well as more patients that had attempted suicide [12]. Moreover, the authors found several cases with possible production of the specific antibody in the central nervous system [13] and a case with first-episode schizophrenia whose specific antibody titers greatly increased during the first 5 weeks of admission [14]. These findings are indicative of the transmission of BoDV-1 to humans as well as its pathogenicity. While no standardized methods for the detection of specific antibodies and viral RNA in blood have been established and although the association between infection and psychiatric disorders remains unclear, four cases of acute fatal encephalitis caused by BoDV-1 reported in Germany in 2018 demonstrate that the virus infects humans [15, 16].

Here, we used a previously developed assay involving a virus-specific antibody and radioligand [17, 18]; this approach is highly sensitive and specific because the radioisotope-labeled antigen, which is suspected to retain its three-dimensional structure, reacts with serum in the liquid phase. In a recent report, antibodies against SARS-CoV-2 nucleocapsid proteins were measured using this method, and the results were in good agreement with the participant's infection history as well as with the results measured by a commercial chemiluminescence immunoassay [19].

Ribavirin, in combination with interferon, is a broad-spectrum antiviral agent approved in Japan since 2001 for the treatment of hepatitis C. Ribavirin reportedly inhibits BoDV-1 in vitro and in vivo [20–22]. Although hemolytic anemia is a common side effect of ribavirin treatment and caution should be exercised given the risk of teratogenicity, it is unlikely to cause any other serious side effects. A previously reported clinical trial of oral ribavirin in seropositive patients with refractory neuropsychiatric symptoms showed clear improvement in two of nine patients [23]. Here, we report the case of a patient with a medical history of severe schizophrenia for more than 30 years who showed some improvement after ribavirin treatment. This therefore represents a unique case of schizophrenia that responded to antiviral treatment.

## 2. Patient and Methods

This study represents the off-label use of ribavirin for the treatment of mental diseases and was approved by the Certified Review Board of Osaka General Medical Center as a specific clinical study according to national regulations (approval number: jRCTs051180130). Ribavirin was orally administered as a single agent according to the regimen for hepatitis C. The patient was recruited from among those who visited the medical center and consented to participate in a study on BoDV-1 infection, which was likewise approved by the ethics committee of the Osaka General Medical Center (approval number: 28-C0202).

Antibody measurements against BoDV-1 were performed using a radioligand assay as previously described [18, 19], while the Brief Psychiatric Rating Scale (BPRS) was used to assess psychiatric symptoms. The patient's family provided written informed consent for publication of this case on her behalf.

## 3. Case

### 3.1. Case History

The patient was a 50-year-old Japanese woman with no family history of psychiatric illness. She had no perinatal or childhood developmental abnormalities. Her demeanor toward others throughout her school and adolescent years was reportedly kind and cheerful, with a strong sense of injustice and a good relationship with family and friends.

**Figure 1:**
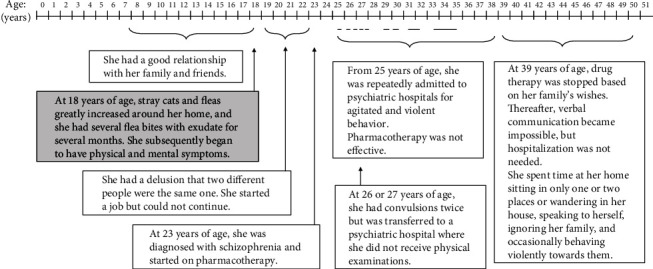
The patient's clinical history. Horizontal bars indicate hospitalizations.

Figure 1 shows the time course of the patient's medical history. When the patient was 18 years old, stray cats started gathering in the basement of her housing complex, resulting in a flea infestation in her home during the summer months. The patient was persistently bitten for several months, resulting in more than 20 flea bites with exudate at the same time during the peak period. She experienced physical symptoms such as low-grade fever, headache, constipation, vomiting, diarrhea, blocked throat, and coldness, as well as mental and neurological symptoms such as insomnia, poor concentration and memory, sudden unexplained sobbing, excessive yawning, decreased frequency of speech, infant-like behavior, and staggering. Additionally, she once lost consciousness for several minutes without experiencing convulsions. One year later, an incident occurred where she strongly insisted that two different people were the same person. At the age of 20 years, she found employment but was unable to retain it due to the inability to perform the job.

At the age of 23 years, she was diagnosed with schizophrenia and started on pharmacotherapy. At 25 years, she was first admitted to a psychiatric hospital after police officers apprehended her for walking on a motorway. She was laughing excessively and exhibited disorganized thinking and violent behavior. She twice had convulsions at the age of either 26 or 27 years but was transferred to a psychiatric hospital where she did not undergo physical examination. By the age of 35 years, she had been hospitalized nine times for acute psychotic episodes involving agitation and violent behavior.

The administered medication did not improve the patient's mental symptoms. Her family noticed that changing the medication sometimes exacerbated her symptoms. At the age of 39 years, her family decided to taper and discontinue her medication, after which she stopped speaking, except for soliloquies. However, her confusion and agitation gradually decreased, which made it easier for the family to cope. For the next decade, she was unmedicated, would only sit in a few places or sporadically wander around inside the house, talk to herself, ignore her family, and occasionally commit acts of violence against them as an exaggerated reaction to trivial stimulation. The patient's family had consulted several hospitals over the previous 3 years to enquire about anti-N-methyl-D-aspartate (NMDA) receptor antibody testing; however, their requests were not met. The family then learned about our clinical trial on the internet and contacted us.

First, only a family member was interviewed about the patient's condition. According to this relative, five other people in the same community had begun to exhibit mental health problems almost simultaneously. This included the patient's sister, who felt less able to concentrate and was unable to make decisions for several months. A 20-year-old daughter of another family became emotionally unstable and ultimately died of a drug overdose; her mother was also acting inappropriately. An elderly man and woman from two other families were diagnosed with depression.

The physician decided to admit the patient to the hospital overnight for a thorough examination.

### 3.2. Hospitalization for Examination

The patient arrived at our hospital on a stretcher. She was lying in a prone position, and her face had a stiff expression. She was not well-kept, stiffened when touched, and did not speak. The examination procedures were explained to the patient in the presence of her family. Several staff members had to hold the patient's body during the blood sampling. Spinal fluid analysis and imaging were performed under sedation.

**Figure 2:**
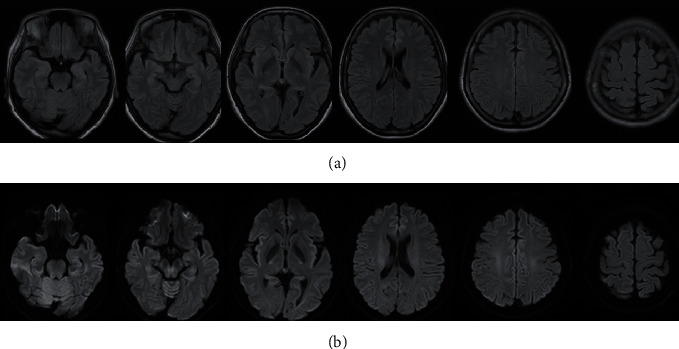
Cranial magnetic resonance imaging findings. Fluid-attenuated inversion recovery imaging (a) and diffusion-weighted imaging (b) at 2 months before treatment.

The standard blood test results revealed no abnormalities. Blood thyroid hormone levels were normal and test results for antinuclear, anti-dsDNA, anti-SS-A, and SS-B antibodies were negative. Infection-related test results were negative, as were the test results for blood anti-Toxoplasma IgG, IgM, and anti-herpes simplex IgG. Cerebrospinal fluid tested negative for anti-NMDA receptor antibodies and showed no abnormalities, with 3/3 mm^3^ cells and 21 mg/dL of protein. Computed tomography of the trunk revealed no ovarian tumors or other abnormalities, and magnetic resonance imaging of the brain revealed no signs of atrophy (Figure 2).

After the patient's discharge from hospital, her IgG and IgM antibodies against BoDV-1 N and BoDV-1 P were assessed, revealing positive results for IgG against BoDV-1 N. Her antibody levels against BoDV-1 P were also slightly high but below the threshold.

### 3.3. Ribavirin Therapy

The family was informed that the patient had antibodies against BoDV-1; however, the levels were not notably high. It was made clear that an antiviral drug could not be expected to show a marked effect at 30 years from disease onset and that unexpected changes, including worsening of symptoms, might occur. However, the family still desired treatment; written informed consent was therefore obtained, and treatment was initiated.

Ribavirin inevitably causes hemolytic anemia, and it can be assumed that a moderate decrease in hemoglobin concentration allows ribavirin to reach effective blood levels [24]. For safety reasons, drug administration was started at 400 mg/day, 200 mg lower than the dose used to treat hepatitis C. As there was no decrease in the patient's blood hemoglobin levels at 4 weeks, the dose was increased to 600 mg/day. Two weeks later, her blood hemoglobin level had decreased from 12.5 g/dL to 11.2 g/dL. Eight weeks after the start of the treatment, the family reported that while she used to be completely indifferent to her family, they had started to observe subtle reactions to them, such as her eyes following someone, and that they had made eye contact with her several times. Similar observations were later reported, but there was no further improvement, and the treatment was terminated at 24 weeks. No other drugs were administered to the patient during or after the treatment.

### 3.4. Changes after Completion of Ribavirin Treatment

Eight weeks post treatment, the patient's family reported that her facial expressions and movements seemed slightly calmer than before, but she was still violent when they came close to her.

Seven months post treatment, her family additionally noticed that the “ominous soliloquies” that she used to frequently utter had not been heard since the end of the ribavirin treatment. These monologues were related to death and often included mentions of “three dead bodies” and “the grave,” as well as the statement “I am surely dead since long ago.” These soliloquies had been repeated approximately once a week over the course of the previous 10 years.

One year after completing the treatment, the patient voiced her elder sister's first name twice and asked her a question using a proper sentence (although her phrasing was fanciful), which had rarely been heard from her for many years. Previously, she would stiffen immediately when her family approached her, but she now appeared less nervous and seemed to tolerate them coming close to her. The threat of her violent behavior toward others, which had always been hanging over the family, greatly lessened.

The patient's BPRS score was 71 before the start of ribavirin treatment and remained unchanged; the psychiatrist could not see any signs of improvement. Seven and 12 months after the treatment, her scores were 66 and 64, respectively, estimated on the basis of family reports because the patient did not visit the hospital.

## 4. Discussion

The patient exhibited significant impairments in social functioning, thought deterioration, delusions, and hallucinations, consistent with the DSM-5 criteria for schizophrenia. However, she also had symptoms suggestive of organic neurological diseases, such as memory impairment and seizures with loss of consciousness. Delusional misidentification can also occur in patients with organic brain lesions [25]. The patient had maintained very good relationships with people before disease onset. However, she engaged in occasional acts of violence after developing psychosis. This discrepancy is often observed in organic neurological diseases. Based on the patient's history and our examination results, substance use-related disorders, paraneoplastic neurological syndromes, and autoimmune diseases such as systemic lupus erythematosus were ruled out.

The changes noticed by her family members during the treatment might have been the result of the family's hope for improvement and their eagerness to find even subtle signs. We were not able to confirm these changes objectively. However, the reported disappearance of the “ominous monologues” 7 months post treatment seems to be an objectively recognizable change and one that is suggestive of an improvement in the patient's psychotic symptoms. The delusion “I am dead” is a symptom of Cotard's syndrome, a severe psychotic condition in which a patient is completely disconnected from reality. Overcoming this severe mental state was indeed a positive change for our patient. Other changes observed later also indicated an improvement in her mental state.

The patient's ailments were originally triggered by flea bites resulting from a cat-related flea infestation. At least five other people in the neighborhood experienced mental health problems at the same time. Although this may have been coincidental, it is also possible that the six individuals' illnesses were related.

Torrey and Yolken reported that contact with cats might be a risk factor for schizophrenia [26], and Toxoplasma infection has been associated with some cases of schizophrenia [27]. According to a recent report, the combination of a Val105/158Met variation in catechol-O-methyltransferase and a Toxoplasma infection significantly increases the risk of schizophrenia, strongly supporting the association between them [28]. In the present case, however, the patient tested negative for anti-Toxoplasma antibodies. BoDV-1 is known to infect cats [29]. *Coxiella burnetii*, *Corynebacterium ulcerans*, *Capnocytophaga canimorsus*, *Pasteurella*, and *Leptospira* can also be transmitted to cats but are unlikely to cause chronic neuropsychiatric symptoms.

Two routes of BoDV-1 transmission via neurons have been demonstrated. The primary route of infection is via exposed olfactory nerve in the nasal mucosa, which encounters viruses present in the nasal or salivary secretion from infected animals; the virus moves up the exposed nerve into the central nervous system [30]. The other route involves transmission from skin wounds through exposed peripheral nerve fibers and into the brain, which takes several weeks [31]. Transmission via fleas has not been confirmed; however, the peripheral nerves exposed to flea bites could transmit the virus to the central nervous system. Contact transmission could occur after touching flea bites or one's nose with a hand contaminated with the virus excreted from cats.

The question is whether the persistence of severe psychotic symptoms in the case described here was due to the virus or a chronic inflammatory response. Jacomb et al. have suggested that a peripheral blood C-reactive protein concentration of 3 mg/L during the acute phase may distinguish high from low “inflammatory biotypes” [32]. In the present case, C-reactive protein concentrations ranged between 0.2 and 1.3 mg/L throughout the treatment period. Although these data were not obtained during the acute phase of the disease, our patient does not fit the typical definition of the high inflammatory biotype. However, this does not exclude the possibility of the involvement of immune dysfunction.

Kamitani et al. [33] observed increased aggression, impaired spatial memory, and decreased serotonin receptor expression in transgenic mice expressing BoDV-1 P in glial cells, but no inflammatory response or degeneration of brain tissue [33]. This indicates that BoDV-1 can induce neuropsychiatric symptoms independent of an inflammatory response.

BoDV-1 is believed to be capable of latent persistent infection and reactivation; however, the duration of the infection remains unclear. Heinrich and Adamaszek [34] examined titers of anti-BoDV-1 antibodies measured using indirect immunofluorescence assay in psychiatric patients who had been tested repeatedly and showed that the titers in patients with schizophrenia in the advanced phase tended to be higher than those in the early phase. They speculated that BoDV-1 infections might be persistent in patients with schizophrenia who consistently have specific antibodies. The presence of disease-specific antibodies in our case may indicate prolonged or repeated antigenic stimulation. If the virus was still present in the patient's brain 30 years after the onset of the disease, and if ribavirin suppressed the virus, it is possible that the psychotic symptoms in this patient were caused by BoDV-1.

The specific serum antibody levels at the end of the ribavirin administration were similar to those before treatment. However, in one previously reported case that was responsive to ribavirin, a decrease in antibody levels was observed 2 years after the end of treatment [23].

The limitations of this study are as follows: first, this is a single case report, and the reproducibility of the treatment effect and frequency of similar cases are unknown. Second, the association between psychiatric disease and BoDV-1 in this case was based on indirect evidence, that is, on the presence of antibodies and changes observed with ribavirin treatment. Since ribavirin suppresses many viruses, the possible involvement of viruses other than BoDV-1 cannot be ruled out. However, unlike that of fatal encephalitis, it is difficult to directly determine the pathogenicity of viruses that persist in the brain. Third, the evaluation of symptoms in this case was based mainly on family observations and the judgment of the physician in charge; findings were not confirmed by a third party.

In conclusion, our findings do not confirm the etiological involvement of infectious agents in this case but suggest that viral agents including BoDV-1 may cause severe schizophrenic symptoms. Since persistent infection with this virus causes behavioral changes in animals, it would be unreasonable to suggest that BoDV-1 does not affect human health. Further studies are required to elucidate the effects of persistent BoDV-1 infections in humans.

## Data Availability

Data supporting the findings of this study are available from the corresponding author upon reasonable request.

## References

[1] Fillman S. G., Weickert T. W., Lenroot R. K. (2016). Elevated peripheral cytokines characterize a subgroup of people with schizophrenia displaying poor verbal fluency and reduced Broca's area volume. *Molecular Psychiatry*.

[2] Zhang Y., Catts V. S., Sheedy D., McCrossin T., Kril J. J., Shannon Weickert C. (2016). Cortical grey matter volume reduction in people with schizophrenia is associated with neuro-inflammation. *Translational Psychiatry*.

[3] Corvin A., Morris D. W. (2014). Genome-wide association studies: findings at the major histocompatibility complex locus in psychosis. *Biological Psychiatry*.

[4] Schizophrenia Working Group of the Psychiatric Genomics Consortium (2014). Biological insights from 108 schizophrenia-associated genetic loci. *Nature*.

[5] Arias I., Sorlozano A., Villegas E. (2012). Infectious agents associated with schizophrenia: a meta-analysis. *Schizophrenia Research*.

[6] Torrey E. F., Bartko J. J., Lun Z. R., Yolken R. H. (2007). Antibodies to Toxoplasma gondii in patients with schizophrenia: a meta-analysis. *Schizophrenia Bulletin*.

[7] Azami M., Jalilian F. A., Khorshidi A., Mohammadi Y., Tardeh Z. (2018). The association between Borna disease virus and schizophrenia: a systematic review and meta-analysis. *Asian Journal of Psychiatry*.

[8] Kinnunen P. M., Palva A., Vaheri A., Vapalahti O. (2013). Epidemiology and host spectrum of Borna disease virus infections. *The Journal of General Virology*.

[9] Hornig M., Solbrig M., Horscroft N., Weissenböck H., Lipkin W. I. (2001). Borna disease virus infection of adult and neonatal rats: models for neuropsychiatric disease. *Current Topics in Microbiology and Immunology*.

[10] Amsterdam J. D., Winokur A., Dyson W. (1985). Borna disease virus: a possible etiologic factor in human affective disorders?. *Archives of General Psychiatry*.

[11] Bechter K., Herzog S., Schüttler R. (1992). Possible significance of Borna disease for humans. *Neurology Psychiatry and Brain Research*.

[12] Bechter K., Herzog S., Estler H. C., Schüttler R. (1998). Increased psychiatric comorbidity in Borna disease virus seropositive psychiatric patients. *Acta Psychiatrica Belgica*.

[13] Bechter K., Herzog S., Behr W., Schüttler R. (1995). Investigations of cerebrospinal fluid in Borna disease virus seropositive psychiatric patients. *European Psychiatry*.

[14] Bechter K., Herzog S., Schreiner V., Wollinsky K. H., Schüttler R., Müller N. (1999). Cerebrospinal fluid filtration in a case of schizophrenia relate to “subclinical” Borna disease virus encephalitis. *Psychiatry, Psychoimmunology, and Viruses*.

[15] Korn K., Coras R., Bobinger T. (2018). Fatal encephalitis associated with Borna disease virus 1. *The New England Journal of Medicine*.

[16] Schlottau K., Forth L., Angstwurm K. (2018). Fatal encephalitic Borna disease virus 1 in solid-organ transplant recipients. *The New England Journal of Medicine*.

[17] Matsunaga H., Tanaka S., Sasao F. (2005). Detection by radioligand assay of antibodies against Borna disease virus in patients with various psychiatric disorders. *Clinical and Diagnostic Laboratory Immunology*.

[18] Matsunaga H., Tanaka S., Fukumori A. (2008). Isotype analysis of human anti-Borna disease virus antibodies in Japanese psychiatric and general population. *Journal of Clinical Virology*.

[19] Matsunaga H., Makino A., Kato Y. (2021). Radioligand assay-based detection of antibodies against SARS-CoV-2 in hospital workers treating patients with severe COVID-19 in Japan. *Viruses*.

[20] Mizutani T., Inagaki H., Araki K., Kariwa H., Arikawa J., Takashima I. (1998). Inhibition of Borna disease virus replication by ribavirin in persistently infected cells. *Archives of Virology*.

[21] Solbrig M. V., Schlaberg R., Briese T., Horscroft N., Lipkin W. I. (2002). Neuroprotection and reduced proliferation of microglia in ribavirin-treated bornavirus-infected rats. *Antimicrobial Agents and Chemotherapy*.

[22] Lee B. J., Matsunaga H., Ikuta K., Tomonaga K. (2008). Ribavirin inhibits Borna disease virus proliferation and fatal neurological diseases in neonatally infected gerbils. *Antiviral Research*.

[23] Matsunaga H., Fukumori A., Mori K., Honda T., Uema T., Tomonaga K. (2018). Two neuropsychiatric cases seropositive for Bornavirus improved by ribavirin. *Japanese Journal of Infectious Diseases*.

[24] Lindahl K., Schvarcz R., Bruchfeld A., Stahle L. (2004). Evidence that plasma concentration rather than dose per kilogram body weight predicts ribavirin-induced anaemia. *Journal of Viral Hepatitis*.

[25] Darby R. R., Laganiere S., Pascual-Leone A., Prasad S., Fox M. D. (2017). Finding the imposter: brain connectivity of lesions causing delusional misidentifications. *Brain*.

[26] Torrey E. F., Yolken R. H. (1995). Could schizophrenia be a viral zoonosis transmitted from house cats. *Schizophrenia Bulletin*.

[27] Torrey E. F. (2022). *Parasites, Pussycats and Psychosis: The Unknown Dangers of Human Toxoplasmosis*.

[28] Rovira P., Gutiérrez B., Sorlózano-Puerto A. (2022). Toxoplasma gondii seropositivity interacts with catechol-O-methyltransferase Val105/158Met variation increasing the risk of schizophrenia. *Genes (Basel)*.

[29] Wensman J. J., Jäderlund K. H., Holst B. S., Berg M. (2014). Borna disease virus infection in cats. *Veterinary Journal*.

[30] Morales J. A., Herzog S., Kompter C., Frese K., Rott R. (1988). Axonal transport of Borna disease virus along olfactory pathways in spontaneously and experimentally infected rats. *Medical Microbiology and Immunology*.

[31] Carbone K. M., Duchala C. S., Griffin J. W., Kincaid A. L., Narayan O. (1987). Pathogenesis of Borna disease in rats: evidence that intra-axonal spread is the major route for virus dissemination and the determinant for disease incubation. *Journal of Virology*.

[32] Jacomb I., Stanton C., Vasudevan R. (2018). C-reactive protein: higher during acute psychotic episodes and related to cortical thickness in schizophrenia and healthy controls. *Frontiers in Immunology*.

[33] Kamitani W., Ono E., Yoshino S. (2003). Glial expression of Borna disease virus phosphoprotein induces behavioral and neurological abnormalities in transgenic mice. *Proceedings of the National Academy of Sciences*.

[34] Heinrich A., Adamaszek M. (2010). Anti-Borna disease virus antibody responses in psychiatric patients: long-term follow up. *Psychiatry and Clinical Neurosciences*.

